# Magic Effects of Hypodermic Puncture of Morphia in Cases of Dysentery

**Published:** 1878-09-20

**Authors:** 


MAGIC EFFECTS OF HYPODERMIC PUNCTURE OF MORPHIA IN CASES OF DYSENTERY.

Dr. J. E. Washington, of Augusta, Georgia, in the Nashville *Journal of Medicine and Surgery*, says:

As I have never seen mention made of the use of morphine by hypodermic puncture in cases of dysentery, I have concluded to give my own experience with it. I was first induced to try it by being called to a case in which there was terrible suffering from tenesmus and vomiting. In this case, the man begged me, "Doctor, for God's sake give me something to relieve me, for I can't stand it much longer." He was covered from head to foot with cold, clammy sweat, lips blue and cold. I gave him about the third of a grain of morphia by puncture—not with any idea that it would relieve the vomiting and purging, but solely to obtund him to the severe suffering; but, to my surprise, in a few moments he was perfectly quiet, and the vomiting and purging almost entirely relieved; another puncture did the work, and he was convalescent in a little over forty hours.

Having such success in this case, I was emboldened to try it in several other cases with equally as good results. After having treated a number of cases in this way, I was taken with an attack of dysentery myself. In my own case, there was severe vomiting and tenesmus—in fact, a movement from the bowels every five or ten minutes, and sometimes I could not leave the stool more than three or four steps without having to return. I tried opium to quiet me, but could not retain it. I then thought of the hypodermic puncture, and, although so weak and faint that I could not sit up, prepared the instrument (being alone) and gave myself a good puncture, and, lo! in a few minutes, I was perfectly relieved. I then applied a wet bandage over stomach and bowels, and was soon convalescent.

Now, here we have, not only the evidence of the beneficial effects of hypodermic puncture of morphine in cases of dysentery, as derived from a trial upon others, but also from personal experience. When we come to consider the severe, debilitating effects of this disease, and also how frequently its effects are prolonged for days and weeks, it behooves us to try those remedies which will cut short the duration of the disease.

I give these ideas for what they may be worth, and would be glad if some of your readers would try the above plan, and see what is the result in their hands.

No more at present; will send you an account of cases of chronic diarrhœa treated and cured by alkaline treatment.

---

TREATMENT OF CANCER OF THE BREAST.

Z. H. Evans, M. D., (*Toledo Medical and Surgical Journal*, April, 1878), treats cancer of the breast by the knife, supplemented by the free use of supersulphate of zinc (Tanner), to accomplish two indications: First, to arrest hemorrhage; and, second, to destroy any of the cancer cells that may remain after the knife. He then has an open wound to treat, which is allowed to contract and cicatrize.—*Detroit Lancet.*

